# Mycophenolate suppresses inflammation by inhibiting prostaglandin synthases: a study of molecular and experimental drug repurposing

**DOI:** 10.7717/peerj.11360

**Published:** 2021-04-30

**Authors:** Fahad Al-Hizab, Mahmoud Kandeel

**Affiliations:** 1 Department of Pathology, College of Veterinary Medicine, King Faisal University, Alahsa, Saudi Arabia; 2 Department of Biomedical Sciences, College of Veterinary Medicine, King Faisal University, Al-Ahsa, Saudi Arabia; 3 Department of Pharmacology, Faculty of Veterinary Medicine, Kafrelsheikh University, Kafrelsheikh, Egypt

**Keywords:** Mycophenolate, Inflammation, Cyclooxygenase, Drug repurposing

## Abstract

Mycophenolate mofetil is an established anti-proliferative and immune-suppressive agent that minimizes the proliferation of inflammatory cells by interfering with nucleic acid synthesis. Herein, we report our discovery of the prostaglandin inhibiting properties of MMF, which offers new applications for the drug in the treatment of inflammatory diseases. The estimated values of IC_50_MMF_COX-1_, IC_50_MMF_COX-2_, and IC_50_MMF_5-LOX_ were 5.53, 0.19, and 4.47 µM, respectively. In contrast, mycophenolic acid (MPA) showed slightly stronger inhibition: IC_50_MPA_COX-1_, IC_50_MPA_COX-2_, and IC_50_MPA_5-LOX_ were 4.62, 0.14, and 4.49 µM, respectively. These results indicate that MMF and MPA are, respectively, 28.6 and 33 times more selective for cyclooxygenase-2 than for cyclooxygenase-1, which implies that MMF would have less impact on the gastric mucosa than most nonselective, nonsteroidal anti-inflammatory drugs. Furthermore, MMF provided dose-dependent relief of acute inflammation in the carrageenan-induced rat paw edema test, with results comparable to those of celecoxib and indomethacin. Molecular dynamics simulations indicated that the MMF bond with COX-2 was stable, as evidenced by a low root-mean-square deviation of atomic positions, complementary per-residue root-mean-square fluctuation, and 0–4 hydrogen bonds during the 50-ns simulation time. Therefore, MMF provides immune-suppressing, cyclooxygenase-inhibiting, and inflammation-relieving properties. Our results indicate that MMF can be 1) repositioned for inflammation treatment without the need for further expensive clinical trials, 2) used for local acute inflammations, and 3) used as a sparing agent for other steroid and non-steroid anti-inflammatory medications, especially in topical applications.

## Introduction

**Figure 1:**
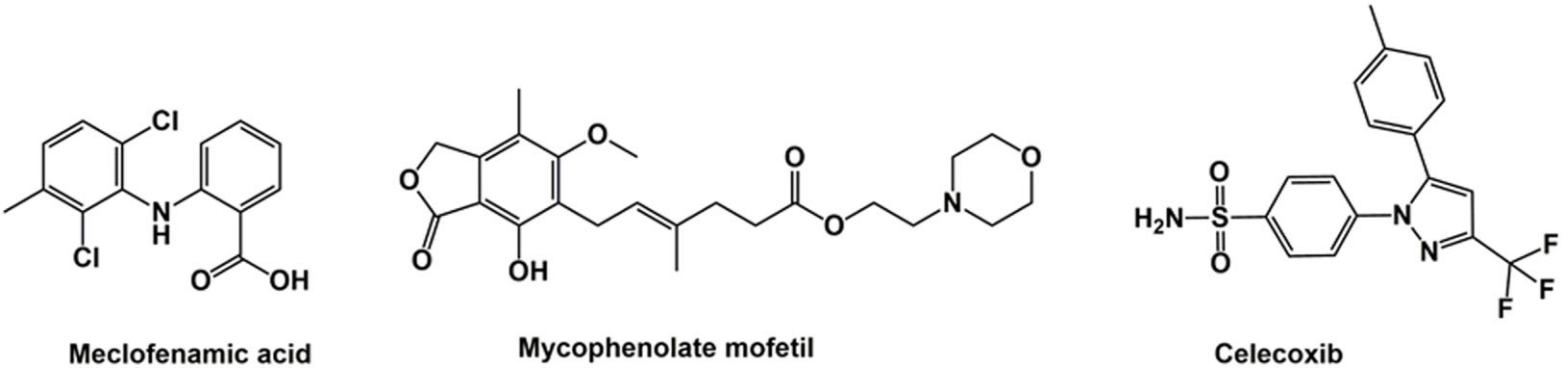
Structure of meclofenamic acid, mycophenolate mofetil and celecoxib.

Mycophenolate mofetil (MMF, Fig. 1) is a nonsteroidal immunosuppressive agent used in treating cancers (Benjanuwattra et al., 2020; Ling et al., 2018), ocular inflammations (Daniel et al., 2010), atopic dermatitis (Grundmann-Kollmann et al., 2001), psoriasis and other dermatological diseases (Orvis et al., 2009), neuromuscular diseases (Chaudhry et al., 2001), organ transplants and autoimmune diseases (Appel, Radhakrishnan & Ginzler, 2005). It is considered a corticosteroid-sparing agent in eye inflammation (Thorne et al., 2005). The advantage of MMF is it is less expensive than nonsteroidal immune-suppressive agents, such as cyclosporine, and has minimal side effects (Daniel et al., 2010). MMF is a prodrug of mycophenolic acid (MPA), an antimetabolite that inhibits inosine monophosphate dehydrogenase and decreases the recruitment of lymphocytes and monocytes into sites of inflammation (Allison & Eugui, 2000). Due to its antimetabolite activity, MMF and its active form (MPA), either alone or in combination with other antimicrobial agents, demonstrate anti-parasitic and antiviral activity (Allison & Eugui, 2000; Margolis et al., 1999). However, MMF replaced MPA in clinical use after the latter’s gastrointestinal side effects were discovered (Park, 2011). Rapidly absorbed after taken orally, MMF is completely converted to MPA by liver carboxylesterases 1 and 2 (Lamba et al., 2014), and maximum serum MPA concentration is attained within 60–90 min of MMF administration (Park, 2011).

NSAIDs are effective in managing acute inflammation. However, the long-term use of NSAIDs is associated with several gastrointestinal, hepatic, renal, and cardiac side effects (Curiel & Katz, 2013; Melcarne, García-Iglesias & Calvet, 2016). Thus, drugs with additional anti-inflammatory actions can preempt NSAID use during treatment, thereby minimizing the need for drug combinations and preventing multiple potential side effects.

NSAIDs act via inhibition of COX-1 and COX-2 activity, resulting in decreased production of prostaglandin E2 (PGE2) (Lacroix & Rivest, 1998). COX-2 is induced by cytokines, lipopolysaccharides, and other inflammatory mediators at the site of inflammation. It mediates the release of PGE2 using arachidonic acid as a substrate, which assists in exudate release and swelling in the inflammatory processes (Morita, 2002). Inhibition of COX-2 has been associated with the treatment of swelling and pain associated with acute inflammation (Seibert et al., 1994).

Drug repurposing is a vital step in the drug discovery process that reallocates a drug with specific applications to other therapeutic uses. This strategy has proven to be effective economically and scientifically, in part because it bypasses the long phases of clinical trials. Multiple successful drug-repurposing trials have led to the discovery of new drug applications. Several examples include sildenafil, initially produced to treat angina and later for male erectile dysfunction (Goldstein et al., 1998), zidovudine, from treating cancers to then fighting HIV (Ashburn & Thor, 2004), and dapoxetine, from an antidepressant to later managing premature ejaculation (Fu, Peng & Hu, 2019).

MMF may have enormous potential as an anti-inflammatory drug as it is among the top 10% hits of FDA-approved drugs with possibly strong binding with COX-2 (Supplemental Data). In addition, several previous clinical reports described the effectiveness of MMF in treating inflammations of the eye, skin, and other organs (Appel, Radhakrishnan & Ginzler, 2005; Chaudhry et al., 2001; Daniel et al., 2010; Grundmann-Kollmann et al., 2001; Orvis et al., 2009; Thorne et al., 2005).

The purpose of this study is to 1) evaluate the potential anti-inflammatory actions of MMF, 2) explore MMF’s anti-inflammatory actions via inhibition of cyclooxygenases, and 3) test the effect of MMF in relieving the acute carrageenan-induced edema on rat paws. The anti-cyclooxygenase activity of MMF is determined by in vitro enzyme inhibition assays against COX-1 and COX-2. Assay results will be compared to those of nonselective cyclooxygenase inhibitor indomethacin (Black et al., 1996) and selective inhibitor celecoxib (Francischi et al., 2002). Finally, we will compare the activities of MMF and MFA in enzyme assays.

## Materials and Methods

### Chemicals, reagents, instruments, and software

The highest quality chemicals including λ-carrageenan, indomethacin, diclofenac sodium, celecoxib, MMF, MFA, tris(hydroxymethyl) aminomethane, hydrochloric acid, carboxymethyl cellulose sodium salt (NaCMC), EDTA, phenol, and hematin were purchased from Sigma–Aldrich, Inc. (St. Louis, MO, USA). Quercetin was obtained from APExBIO (Houston, TX, USA). The COX inhibitor screening assay kit and LOX inhibitor screening kit were purchased from Cayman Chemical (Ann Arbor, MI, USA). The vernier caliper was from SMIEC (Shanghai, China). The Schrodinger Maestro package release 2020.1 (Schrodinger, Inc., LLC, New York, USA) was used in compound and protein preparation and docking runs. Groningen Machine for Chemical Simulations (GROMACS) version 2018.8 software was available at (http://www.gromacs.org/). GraphPad Prism version 7 software was purchased from (GraphPad Software, Inc., San Diego, USA).

### Retrieval and optimization of drug structures

The structures of meclofenamic acid (MFA) and MMF (Fig. 1) were retrieved from the PubChem database (https://pubchem.ncbi.nlm.nih.gov/, accessed on February 10, 2020) and stored in SDF format. The structures were imported to Ligprep software (Schrodinger Maestro package) for 3D optimization using OPLS2005 force field at physiological pH.

### COX-2 structure optimization and preparation

The Maestro software package was used to prepare the structure for molecular docking. The protein (PDB ID 5IKQ, https://www.rcsb.org/structure/5IKQ) was corrected for the missing atoms and residues, protonated, optimized at physiological pH, and energy minimized. The OPLS2005 force field was used. The docking grid was generated by forming a grid box of 20 Å around the bound ligand, meclofenamic acid.

### Molecular docking

The high precision (XP) glide docking module of the Schrodinger Maestro package was used for molecular docking. The results were ranked by docking score. The accuracy of results was confirmed by comparing the docking of MFA with the coordinates of the bound ligand in the structure file. The molecular mechanics/generalized Born surface area (MM/GBSA) was calculated from the docked poses.

### Determination of COX-1, COX-2, and 12-LOX inhibition

The inhibitory effects of the drugs MMF and MPA on COX-1 and COX-2 were measured using the COX inhibitor screening assay kit and LOX inhibitor screening kit. The assay was performed according to the manufacturer’s directions and previously described methods (Chowdhury et al., 2008; Lee et al., 2004). Briefly, the reaction buffer (Tris–HCl buffer, pH 8.0) containing 5 mM EDTA, 2 mM phenol, and 1 µM hematin was measured into test tubes. The test drugs were dissolved in dimethyl sulfoxide and added to the tubes to final concentrations of 0.05–200 mM. COX-1 or COX-2 was added to the test tubes and preincubated for 10 min at 37 °C. Arachidonic acid was then added, and the tubes were incubated for 2 min at 37 °C. The enzyme immunoassay was used to measure the concentration of the product prostaglandin F2α (PGF2α). The estimated values were determined after comparison to the predetermined standard curve. The IC_50_ was determined after three different experiments. The selectivity index was calculated from Eq. (1):

(1) $$Selectivity\;index=\frac{IC_{50}COX-1}{IC_{50}COX-2}$$

### Evaluation of anti-inflammatory activity in rats with carrageenan-induced paw edema

The in vivo assay was performed by the rat paw edema inhibition test as previously described (Morris, 2003; Winter, Risley & Nuss, 1962). Forty-nine male adult albino rats weighing 220–180 g were obtained from the Animal Breeding Center at the College of Veterinary Medicine, King Faisal University, and were randomly divided into seven groups (*n* = 7/group). The animal groups included the nontreated control group, carrageenan group, and five groups receiving carrageenan and treated with either indomethacin, celecoxib, or MMF at three doses (12.5, 25, and 50 mg/kg). All rats were allowed free access to food and water. All cages were kept in controlled rooms at 25 °C and exposed to a 12-h light/dark cycle. MMF and two reference drugs, indomethacin and celecoxib, were suspended in 1% NaCMC in normal saline. A 1% carrageenan solution in normal saline was injected into the subplantar area of the right hind paw of rats. One hour before injection of carrageenan, rats were given either indomethacin or celecoxib at a dose rate of 10 mg/kg or MMF at three dose levels of 12.5, 25, and 50 mg/kg by oral administration. A vernier caliper was used to measure the rat paw thickness of all rats in all groups at 0, 1, 2, 3, 4, and 5 h after drug administration. The measurements were blind to avoid measurement bias. The King Faisal University Ethics Committee (approval no. KFU-REC/2020-10-11) approved all animal experiments. Since no samples were taken from rats, there were no anesthesia procedures. The size of the hind paw was only measured in non-anesthetized rats. At the end of the experiment, all rats were euthanized according to the regulations of the King Faisal University Ethics Committee by an overdose of pentobarbital (200 mg/kg IP) and decapitation.

### Molecular dynamics simulations

GROMACS software was used in all molecular dynamics (MD) simulations (Abraham et al., 2015; Van Der Spoel et al., 2005). AMBERFF14SB force field and general AMBER force field (GAFF) were used to handle the ligand parameter, topology, and restraint and the protein, respectively. The COX-2-drug complexes were solvated in a cubic box filled with a single point-charge (SPC) water model. The boundaries of the box were 1.0 nm from the protein to the box edge. The solvated complexes were minimized for 5,000 steps. During water and ion coupling, the heavy atoms of protein and ligand were restrained. The system equilibration comprised 50 ps NVT at 300 K followed by 1 ns NPT ensemble at 300 K. Production stages were extended for 50 ns with NPT ensemble. The constant 1 bar pressure and 300 K temperature were maintained by Parrinello-Rahman and V-rescale thermostat algorithms, respectively. The PME method was used to manage long-range electrostatics. The time step was 2 fs, and snapshots were collected every 10 ps.

### Post-dynamic analysis

The GROMACS MD simulation toolkits performed the post-dynamic trajectory analysis. RMSD and RMSF were calculated by the g_rms and g_rmsf functions. The number of hydrogen bonds and hydrogen bond length were calculated.

### Statistical analysis

The effect of MMF at different doses on relieving rat paw edema was compared with the control and standard NSAID-treated groups. Significant differences were determined by one-way analysis of variance (ANOVA) with Tukey’s post hoc test. All steps were implemented using GraphPad prism software (GraphPad Software, La Jolla, CA, USA).

## Results

### Molecular docking

The results of drug docking with COX-2 are shown in Table 1. The output parameters include the docking scores, ligand efficiencies, and lipophilic and hydrogen bonding interactions. The docking score of MMF was approximately 78% of the score of MFA. This result was supported by favorable hydrogen bonding and electrostatic and hydrophobic interactions. Notably, these parameters were lower than the standard anti-inflammatory ligand, MFA. The generalized Born model and solvent accessibility (MM/GBSA) binding energy and its decomposition (Table 2) had similar profiles to the estimated docking. The binding of MMF showed lower MM/GBSA binding energy as a result of higher hydrogen bonding and lipophilic interaction scores and lower electrostatic forces.

**Table 1:** Extra-precision docking of meclofenamic acid and mycophenolate mofetil with COX-2.

Drug	Docking score	Ligand efficiency	vdw	Coul	HBond
Meclofenamic acid	−10.29	−0.54	−39.64	−9.46	−1.63
Mycophenolate mofetil	−8.12	−0.31	−29.64	−4.21	−0.82

**Table 2:** The molecular mechanics–generalized Born surface area (MM/GBSA) binding energy for the binding of meclofenamic acid and mycophenolate mofetil with COX-2.

Drug	MMGBSA ΔGBind	MMGBSA ΔGBindCoulomb	MMGBSA ΔGBindHbond	MMGBSA ΔGBindLipo	MMGBSA ΔGBindSolv
Meclofenamic acid	−80.13	−7.94	−0.58	−44.03	12.81
Mycophenolate Mofetil	−48.60	−3.37	−1.14	−66.12	21.18

**Figure 2:**
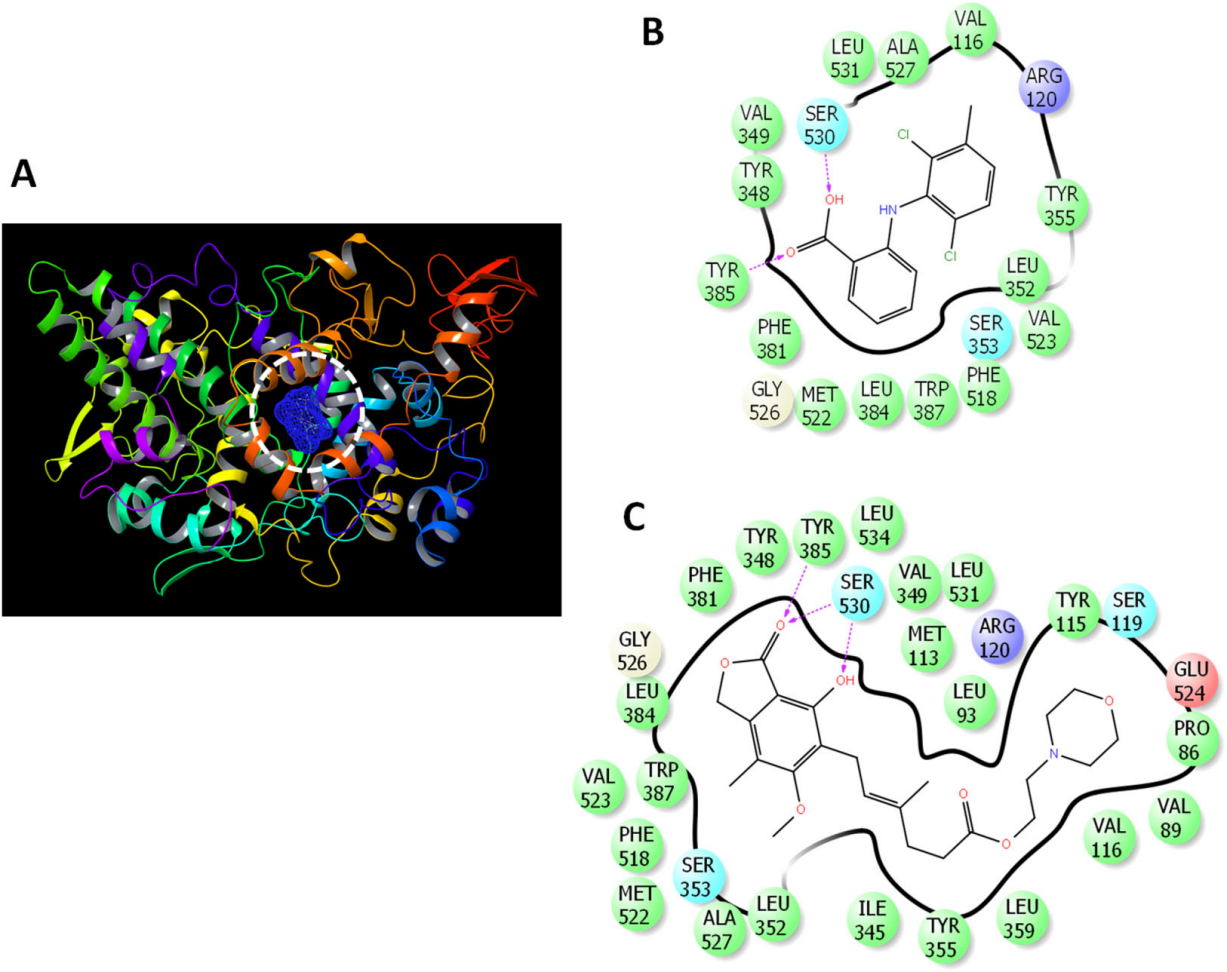
Docking of mycophenolate mofetil into the active site of COX-2. (A) 3D representation of COX-2 showing the active site (enclosed in white dashed circle). (B) The ligand interactions of meclofenamic acid. (C) The ligand interactions of mycophenolate mofetil. Charged residue (negative) in pink, positive charged residue in blue, hydrophobic residues in cyan, hydrogen bonds: purple arrow, stocking interactions: green sticks. The direction of H-bond is towards the acceptor site.

The binding mode was analyzed (Fig. 2) to gain deeper insights into the interaction of drugs with COX-2. MFA formed two hydrogen bonds with TYR385 and SER530 (Fig. 2B). MMF formed similar interaction with the presence of bidentate hydrogen bonds with SER30 (Fig. 2C).

### Inhibition of cyclooxygenases and lipoxygenase by in vitro enzyme assays

The inhibitory potency of MMF and MPA against COX-1, COX-2, and 12-lipooxygenase (12-LOX) was measured and compared to standard inhibitors for these enzymes. MMF and MPA inhibited the three enzymes with MMF IC_50_ values of 5.53, 0.19, and 4.47 µM for COX-1, COX-2, and 12-LOX, respectively. The IC_50_ for MPA was 4.62, 0.14, and 4.49 µM, respectively. The selectivity index for MMF and MPA was at least 29.1 and 6.5-fold higher than indomethacin and diclofenac, respectively. In contrast, it was 11.4-fold lower than celecoxib. Both MMF and MPA were weaker than quercetin in inhibition of 5-LOX with IC_50_ values of 4.47 and 4.49 µM, respectively (Table 3).

**Table 3:** The inhibitory properties of mycophenolate mofetil IC_50_ against COX-1, COX-2 and 12-LOX.

Drugs	IC_50_ (µM)			Selectivity index
	COX-1	COX-2	12-LOX	
Celecoxib	14.7	0.045	ND	326.7
Indomethacin	0.1	0.08	ND	1.3
Quercetin	ND	ND	3.33	–
Diclofenac sodium	3.8	0.84	ND	4.5
Mycophenolate mofetil	5.53	0.19	4.47	28.6
Mycophenolic acid	4.62	0.14	4.49	33.00

**Note:**

IC50 values were compared with standard anti-inflammatory drugs, celecoxib, indomethacin and diclofenac. Celecoxib was used as a reference selective COX-2 inhibitor, two standard non-selective NSAIDs (indomethacin and diclofenac sodium) were used as reference inhibitors. Quercetin was used as selective 12-LOX reference inhibitor.

### Carrageenan-induced paw edema

**Figure 3:**
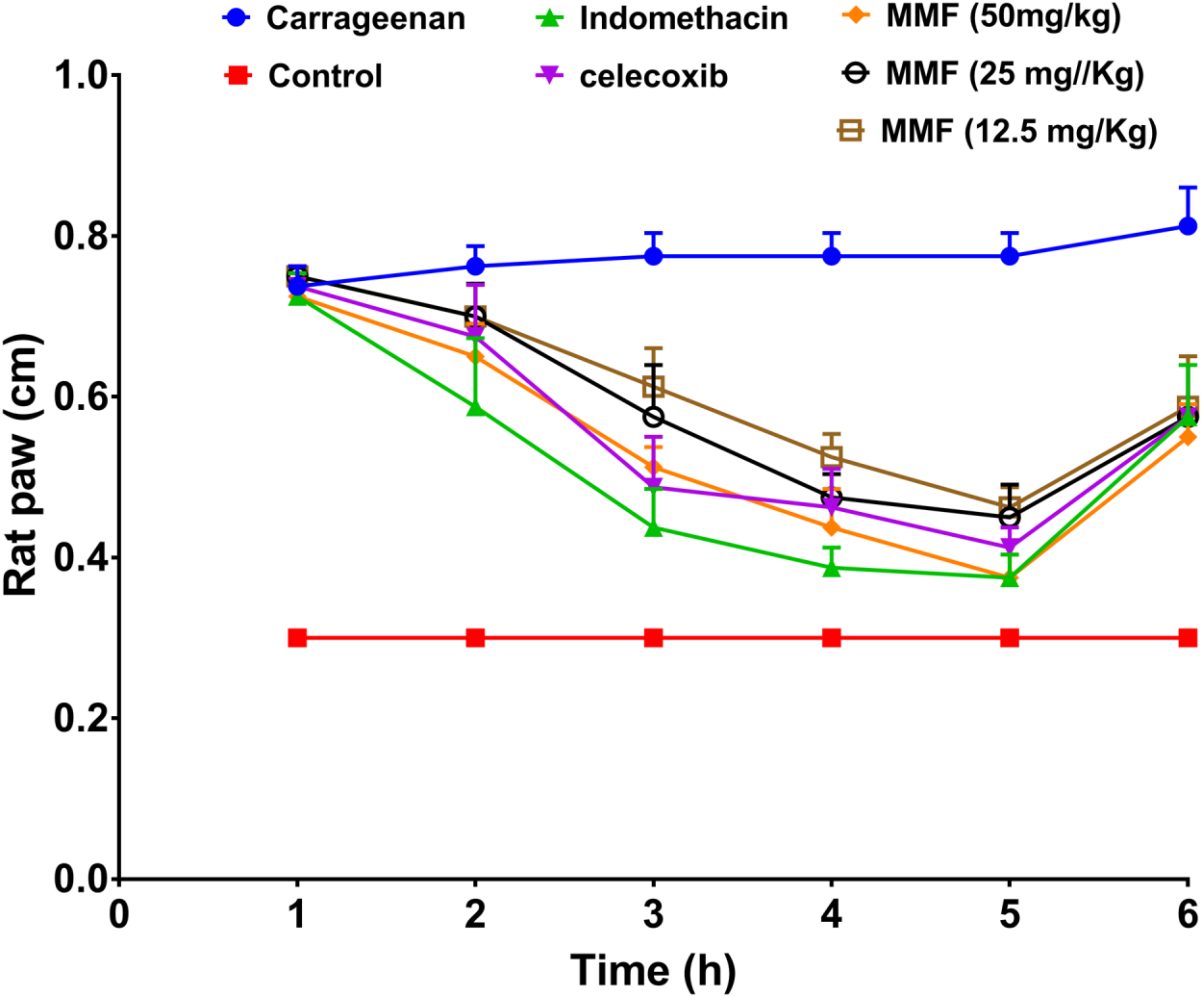
The results of rat paw test for evaluation of the anti-inflammatory activity of mycophenolate mofetil. The *X*-axis shows the time of rat paw size measurement in hours. The *Y*-axis shows the rat paw size in cm. All treatment gave statistically significant relief or rat paw edema, compared with the control non-treated group (*P* < 0.05).

**Figure 4:**
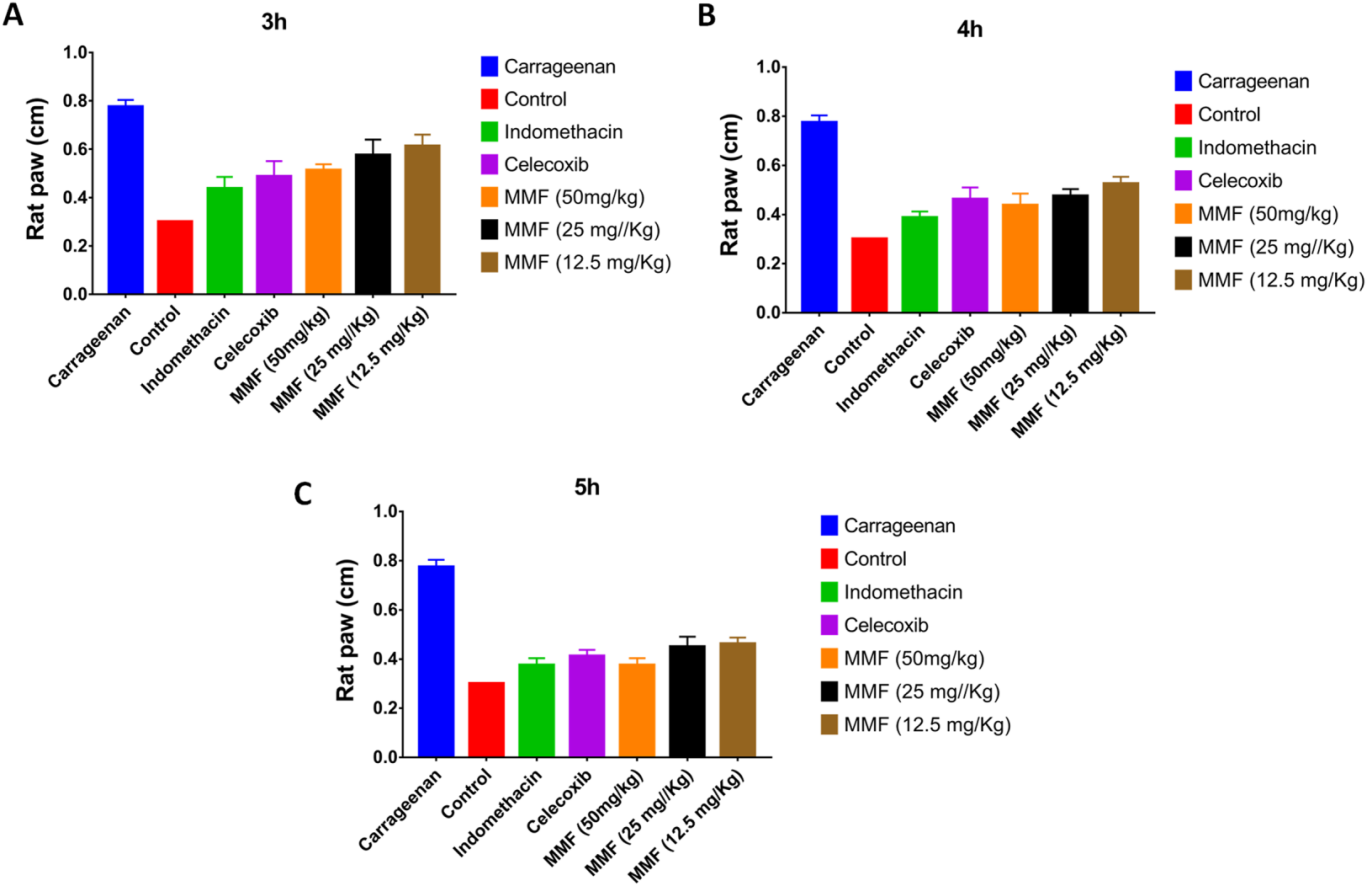
The time course comparison of rat paw response to different treatments. (A) After 3 h of carrageenan injection. (B) After 4 h of carrageenan injection. (C) After 5 h of carrageenan injection. All treatment gave statistically significant relief or rat paw edema, compared with the control non-treated group (*P* < 0.05).

Injection of carrageenan into rat paws resulted in a marked increase in paw size from 0.3 to 0.8 cm. Treatment with 12.5, 25, and 50 mg/kg MMF resulted in a significant decrease in rat paw edema 2–5 h after drug administration (Fig. 3). Measuring rat paw size at 3, 4, and 5 h after treatment revealed a dose-dependent decrease in edema, which was highly comparable to treatment with indomethacin and celecoxib (Fig. 4). Statistical analysis (Table 4) of the treated groups indicated. MMF at 12.5, 25, and 50 mg/kg significantly relieved the rat paw edema (*p* < 0.001) compared to the control group. There was no statistical difference between the effect of MMF and the standard COX inhibitors, indomethacin and celecoxib. Slight differences were observed in the average hourly results in the 3- to 5-h range after treatment (Table 4). Analyzing hours 3 and 4 separately revealed that 50 mg/kg MMF treatment results were significantly different from the other two doses and were comparable to indomethacin and celecoxib. Therefore, the 50 mg/kg dose was the only dose that provided significant relief of paw edema.

**Table 4:** Summary of the statistics significance for comparing the anti-inflammatory actions of mycophenolate mofetil with indomethacin and celecoxib.

Tukey’s multiple comparisons test	Significant?	Summary*	Adjusted *P* value
Carrageenan vs. Control	Yes	s	<0.0001
Comparson of MMF with standard COX inhibitors			
Carrageenan vs. MMF (50 mg/kg)	Yes	s	<0.0001
Carrageenan vs. MMF (25 mg/kg)	Yes	s	0.0001
Carrageenan vs. MMF (12.5 mg/kg)	Yes	s	0.0006
Comparson of MMF with standrd COX inhibitors			
Indomethacin vs. MMF (25 mg/kg)	No	ns	0.2445
Indomethacin vs. MMF (12.5 mg/kg)	No	ns	0.0634
Celecoxib vs. MMF (50 mg/kg)	No	ns	>0.9999
Celecoxib vs. MMF (25 mg/kg)	No	ns	0.9090
Celecoxib vs. MMF (12.5 mg/kg)	No	ns	0.4839

**Note:**

The comparison was based on one-way ANOVA test. The comparison of means was based on the mean values of rat paw test between 3–5 h post carrageenan injection.

* Significant (s) or non-significant (ns)

### Molecular dynamics simulation

**Figure 5:**
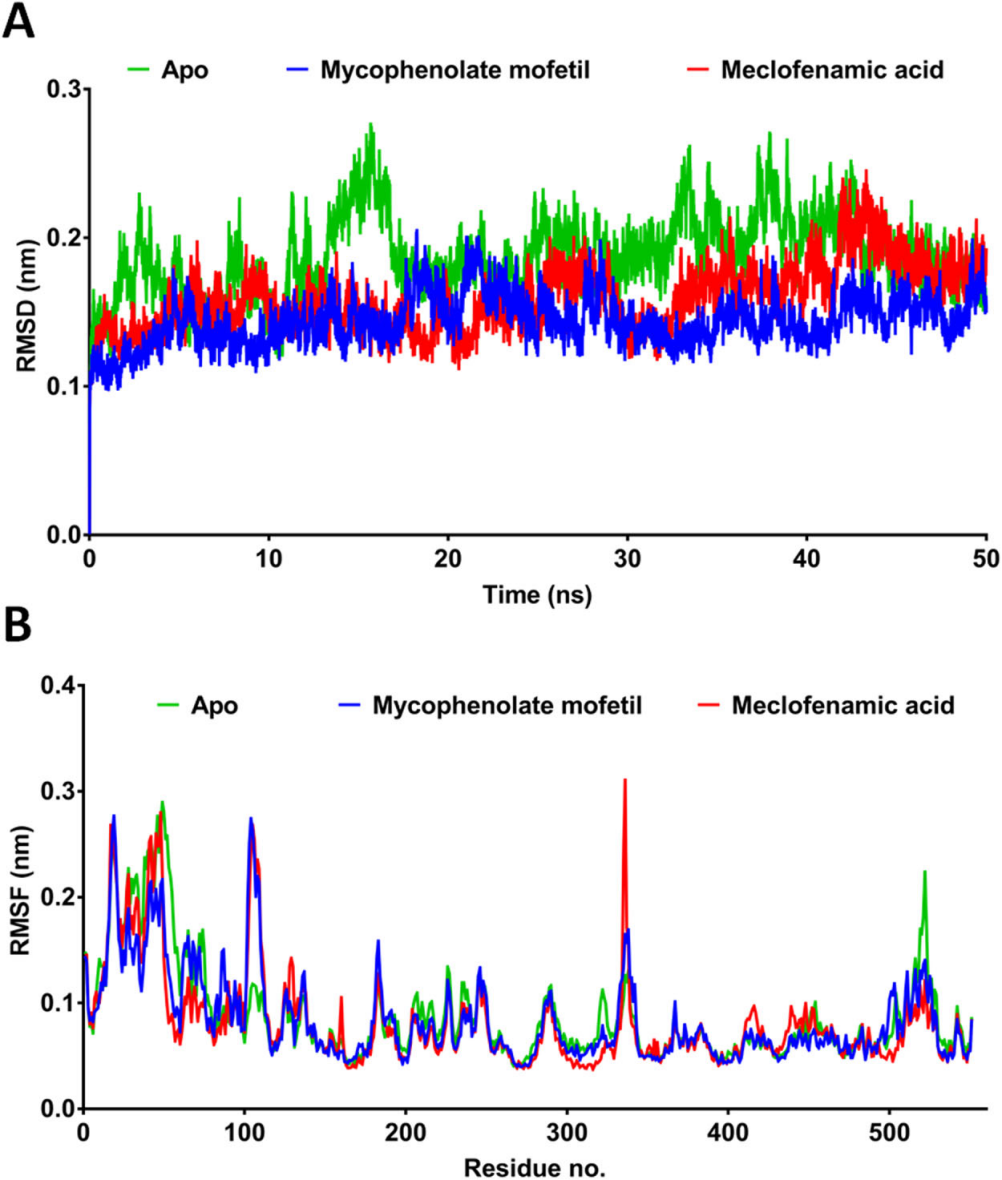
Molecular dynamics simulation for 50 ns. RMSD and RMSF after 50 ns MD simulation for the binding of meclofenamic acid and mycophenolate mofetil with COX-2. (A) RMSD of meclofenamic acid and mycophenolate mofetil. (B) The per-residue RMSF of COX-2 bound with meclofenamic acid and mycophenolate mofetil.

**Figure 6:**
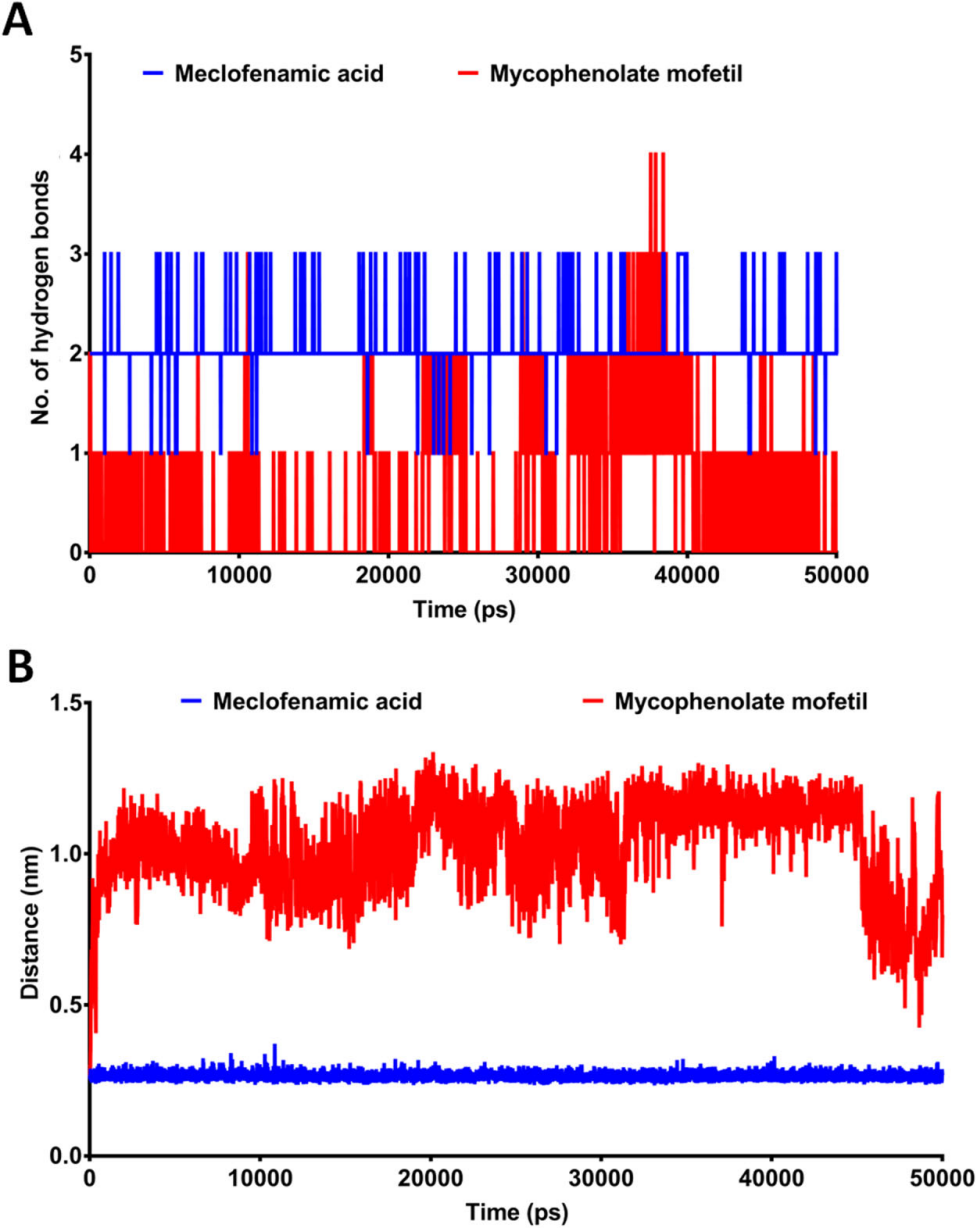
The post-dynamic analysis of MD simulation for 50 ns. (A) The number of hydrogen bonds formed between meclofenamic acid and mycophenolate mofetil and TYR385. (B) The length between MFA and MMF and the residue TYR385 during MD simulation.

MD simulation provides more comprehensive insights into drug-receptor dynamics. These results, in combination with docking runs, help to rank the drugs based on binding affinity and dynamics. Docking followed by MD simulation can provide a complete picture of drug-protein complex stability and structural changes associated with their binding. We subjected ApoCOX-2 or COX-2 bound with MMF and MFA to MD simulations for 50 ns (Fig. 5). The structure RMSD indicated more stable COX-2-drug complexes over the Apo enzyme, indicating stable complexes with the drugs. The drug complexes were evidently stabilized early in the first 5 ns to the end of the simulation. Furthermore, the average RMSD was low (<1.5 Å), indicating good structural stability (Fig. 5A). The RMSF values indicated conserved profiles for all structures with markedly low drug complex values at the C-terminal of COX-2 (Fig. 5B). A high RMSF peak was observed in the residue range 336–338. We traced the number of hydrogen bonds during simulation time (Fig. 6). MFA formed 1–3 hydrogen bonds, while MMF formed 0–4 bonds. In addition, MFA formed a stable and strong H-bond with TYR385 (Fig. 6B). MMF did not show a similarly stable profile with distance ranges from 0.5–1.2 nm. This result might be driven by the high number of rotatable bonds in MMF (11 bonds), compared with three bonds in MFA.

## Discussion

Inflammation comprises the complex interplay between the damaged tissues and inflammatory cells, resulting in the release of inflammatory mediators, including interleukins, necrotic factors, and enzymes such as cyclooxygenases and lipoxygenases (Goldstein, Snyderman & Gallin, 1992). COX-1, COX-2, and 12-LOX upregulation during inflammatory processes plays an essential role in inflammatory signaling pathways (Gunaydin & Bilge, 2018). Control over inflammation is required to avoid excessive damage from the exaggerated immune response or conversion to chronic illness (Yonezawa et al., 2019).

Both steroidal and nonsteroidal anti-inflammatory agents have been clinically approved; however, they face several concerns regarding toxicity (Bjarnason et al., 2018; Eren, Armağan & Talmaç, 2016; Rainsford, 1989). In this study, the anti-inflammatory activity of MMF was compared to that of selective and nonselective COX inhibitors. The inhibitory properties of MMF and MPA against COX-1 were weaker than indomethacin and diclofenac and stronger than celecoxib. Against COX-2, MMF and MPA were weaker than indomethacin and celecoxib and stronger than diclofenac. Celecoxib was the most selective drug, inhibiting COX-2 at a very low concentration, followed by MMF. Therefore, MMF had medium selectivity scores, much lower than celecoxib but higher than the nonselective drugs indomethacin and diclofenac. In addition, MMF/MPA demonstrate 12-LOX IC_50_ values of 4.47/4.49 µM, which was slightly weaker than quercetin. Inhibition of 12-LOX was not observed for the other anti-inflammatory agents examined.

In this study, the carrageenan-induced rat paw swelling test was used to evaluate the anti-inflammatory actions of MMF. This model is ideal for the assay of COX-2 inhibitors, as carrageenan induces exudate release and swelling due to up-regulation of COX-2 expression (Yonezawa et al., 2019). The results of the rat paw’s acute carrageenan-induced inflammation are consistent with the observed in vitro enzymatic assays. The rat paw edema was significantly relieved after treatment with MMF, indomethacin, and celecoxib. In general, the highest dose of MMF was not superior to indomethacin in reducing rat paw edema size. However, there was no statistical significance for the differences between indomethacin and MMF. Rat paw edema proceeds through two stages. The first stage comprises histamine and serotonin release and lasts for 1 h. The second stage extends for several hours and includes the release of inflammatory exudate and enzymes such as cyclooxygenases and cell infiltration (Posadas et al., 2004). Therefore, the observed effect of MMF after 3–5 h of inflammation is consistent with the inhibitory effect on cyclooxygenases. These results are also consistent with previously published reports of the effect of drugs on rat paw edema (Costa et al., 2004). Indomethacin is an ideal drug as a positive control in carrageenan-induced rat paw edema, as it eliminated COX-2 production (Nantel et al., 1999). In this context, MMF produced significant relief of rat paw edema with paw sizes comparable to rats treated with indomethacin.

The estimated IC_50_ of MMF against COX-2 was 0.19 µM, far below the clinically attainable MPA (1–10 µM) (Margolis et al., 1999). This result indicates that the COX-2 inhibitory activity can be attained during the clinical use of MMF. In contrast, the relatively high IC_50_ of MMF against COX-1 (5.53 µM) indicates the lack of potential COX-1 inhibition within the clinical dose range, supporting the protective effect of COX-1 and minimizing the gastrointestinal side effects of MMF. Oral dosage of 1–2 g daily for 12 weeks (Grundmann-Kollmann et al., 2001; Neuber et al., 2000) or topical application of 15% MMF (Handjani et al., 2017) was highly effective in treating atopic dermatitis and vitiligo, respectively. This high topical concentration was well tolerated, suggesting the potential clinical effectiveness of topical MMF as a dual immunosuppressant and anti-inflammatory agent. A high dose after topical use can attain the estimated IC_50_ values of MMF against both COX-1 and COX-2. Although the most effective dose of MMF in the rat paw edema test was 50 mg/kg, such a dose cannot be directly related to human applications and requires further clinical evaluation. MMF could perform at sufficiently high concentrations above the estimated IC_50_ against cyclooxygenases. Recently, new topical formulations such as a micro emulsion-based hydrogel effectively enhanced MMF penetration into the skin (Sharma & Bedi, 2014). Such a formulation could enhance the predicted anti-inflammatory activity of MMF by delivering high concentrations of MMF above the estimated IC_50_ values. The clinical pharmacokinetic estimates of MMF maximal serum concentration (*C*_max_) were approximately 18–20 µg/ml after 1–2 g oral doses (Ferreira et al., 2020). This observation indicates that MMF performs at a *C*_max_ of approximately 3.62 and 105.3 times higher than the IC_50_ values against COX-1 and COX-2, respectively. However, the extent of being above IC_50_ needs further studies. The docking and MD simulation experiments showed that MFA was superior to MMF. However, MMF was able to develop a stable complex with low RMSD and minimal RMSF, while maintaining 0–4 hydrogen bonds during the simulation and stronger hydrophobic attraction. These features contribute to the experimentally estimated values and observation that MMF possesses COX-2 inhibitory properties.

## Conclusions

The results of in silico, in vitro, and in vivo studies suggest that MMF provides substantial anti-inflammatory activity. MMF docking scores and binding profiles with COX-2 suggest its potent inhibitory properties. Experimentally, MMF inhibited COX-1 and COX-2 in the micromolar and nanomolar range, respectively. Moreover, MMF alleviated rat paw edema in a dose-dependent manner. These findings are promising, and further study is needed to evaluate their clinical importance.

## Supplemental Information

10.7717/peerj.11360/supp-1 Supplemental Information 1 Empty COX-2 structure file.

10.7717/peerj.11360/supp-2 Supplemental Information 2 MMF-COX-2 structure file.

10.7717/peerj.11360/supp-3 Supplemental Information 3 Raw data for Apo-COX-2 structure RMSF.

10.7717/peerj.11360/supp-4 Supplemental Information 4 Raw data for MFA-Cox-2 RMSF.

10.7717/peerj.11360/supp-5 Supplemental Information 5 Raw data for MFA-Cox-2 RMSD.

10.7717/peerj.11360/supp-6 Supplemental Information 6 MFA-COX-2 structure file.

10.7717/peerj.11360/supp-7 Supplemental Information 7 Raw data file for MMF-Cox-2 RMSF.

10.7717/peerj.11360/supp-8 Supplemental Information 8 Raw data file for MMF-Cox-2 RMSD.

10.7717/peerj.11360/supp-9 Supplemental Information 9 Raw data for Figures 3 and 4. The rat paw size was measured over time scale from 1-6 hours.

10.7717/peerj.11360/supp-10 Supplemental Information 10 The ARRIVE guidelines 2.0: author checklist.

## Additional Information and Declarations

### Data Availability

The following information was supplied regarding data availability: The Supplemental Files contain the PEERJ ARRIVE 2.0 checklist and raw data files for docking and MD simulation.
